# Evaluation of TEAM dynamics before and after remote simulation training utilizing CERTAIN platform

**DOI:** 10.1080/10872981.2018.1485431

**Published:** 2018-06-18

**Authors:** Kelly M. Pennington, Yue Dong, Hongchuan H. Coville, Bo Wang, Ognjen Gajic, Diana J. Kelm

**Affiliations:** a METRIC Group, Mayo Clinic, Rochester, MN, USA; b Department of Medicine, Division of Pulmonary and Critical Care Medicine, Mayo Clinic, Rochester, MN, USA

**Keywords:** Teamwork, resuscitation, simulation, ICU education, team dynamics

## Abstract

**Objective**: The current study examines the feasibility and potential effects of long distance, remote simulation training on team dynamics.

**Design**: The study design was a prospective study evaluating team dynamics before and after remote simulation.

**Subjects**: Study subjects consisted of interdisciplinary teams (attending physicians, physicians in training, advanced care practitioners, and/or nurses).

**Setting**: The study was conducted at nine training sites in eight countries.

**Interventions**: Study subjects completed 2–3 simulation scenarios of acute crises before and after training with the Checklist for Early Recognition and Treatment of Acute Illness (CERTAIN).

**Measurements and main results**: Pre- and post-CERTAIN training simulations were evaluated by two independent reviewers utilizing the Team Emergency Assessment Measure (TEAM), which is a 11-item questionnaire that has been validated for assessing teamwork in the intensive care unit. Any discrepancies of greater than 1 point between the two reviewers on any question on the TEAM assessment were sent to a third reviewer to judge. The score that was deemed discordant by the third judge was eliminated. Pre- and post-CERTAIN training TEAM scores were averaged and compared. Of the nine teams evaluated, six teams demonstrated an overall improvement in global team performance following CERTAIN virtual training. For each of the 11 TEAM assessments, a trend toward improvement following CERTAIN training was noted; however, no assessment had universal improvement. ‘Team composure and control’ had the least absolute score improvement following CERTAIN training. The greatest improvement in the TEAM assessment scores was in the ‘team’s ability to complete tasks in a timely manner’ and in the ‘team leader’s communication to the team’.

**Conclusion**: The assessment of team dynamics using long distance, virtual simulation training appears to be feasible and may result in improved team performance during simulated patient crises; however, language and video quality were the two largest barriers noted during the review process.

## Introduction

Interprofessional teamwork is associated with patient outcomes in the intensive care unit. Team performance is dependent on technical and nontechnical skills: teamwork, leadership, and task management [1–4]. Clinical experience alone has not been associated with improved team performance [5]; therefore, training programs are focusing on team dynamics during medical crises as a point of improvement. Since team dynamics can be taught and practiced in simulated environments [6,7], various types of simulation have been developed – computer-based, manikin, and standardized patient [2,3,8]. These training tools may not be available in many hospitals and training facilities in low-middle income countries. Internet-based, remote simulation may provide a feasible alternative.

The Checklist for Early Recognition and Treatment of Acute Illness (CERTAIN) is a web-based decision support tool developed for intensive care units in resource variable locations [www.icertain.org] [9]. CERTAIN contains patient relevant information (as an electronic medical record) with hyperlinks to best practice information [9]. For example, if a patient is admitted to the intensive care unit for diabetic ketoacidosis, then information regarding best practice treatment, as dictated by content experts, will be hyperlinked within the CERTAIN platform in that patient’s medical record. This will also occur if the CERTAIN platform flags a lab that is abnormal, such as if a patient has an elevated calcium flagged within CERTAIN, then a hyperlink with evaluation and treatment of hypercalcemia will appear. The primary objective of CERTAIN is to facilitate timely and accurate best practice health-care delivery as a decision support tool. Its secondary objective is to be an interactive educational tool [9]. CERTAIN contains two sections – evaluation of life-threatening emergencies (ELITE) section and a rounding section. Our study focuses on the ELITE section. CERTAIN-ELITE focuses on goal-directed resuscitation. The primary focus of this section is not on team dynamics; however, we believe the simulation provided by CERTAIN-ELITE training and the decision support provided by the tool itself can be utilized to improve team performance.

To objectively assess team effectiveness in emergency situations, several assessment tools have been validated: Mayo High Performance Teamwork Scale [10], Emergency Team Dynamics Scale [1], and Team Emergency Assessment Measure (TEAM) [11]. All three scales have demonstrated reliability and validity [1,10,11]; however, only TEAM focuses on teamwork and leadership in the specific context of medical emergencies. The TEAM tool evaluates team performance on a 0–4 Likert scale in three domains: leadership, team work, and task management [11]. It additionally includes a global assessment of the team’s performance.

Since interprofessional teamwork has been linked with patient outcomes [1] and team dynamics can be improved with simulation [6,7], we set out to determine if team dynamics can be improved via remote simulation in resource-limited areas. The original purpose of the simulations was to assess the effectiveness of CERTAIN-ELITE training; however, we wanted to utilize this pre-existing platform to assess the effects of remote simulation on team dynamics. Utilizing CERTAIN-ELITE and the TEAM tool, we examined the feasibility and potential effects of remote simulation training on team performance during medical emergencies.

## Materials and methods

The study was approved by the Mayo Clinic Institutional Review Board (IRB number 12–007998).

### Study subjects

Interdisciplinary teams at nine training sites in eight countries completed 2–3 simulations of acute crisis before and after training with CERTAIN-ELITE. Training with CERTAIN-ELITE was the hands-on use of the cloud-based, decision support tool guided by remote trainers. Interdisciplinary teams were comprised of attending physicians, physicians in training, advanced care practitioners, and/or nurses depending on training site. The number of team members ranged from three to five individuals. Included sites were Bosnia, Brazil, India, Ireland, Mexico, Saudi Arabia, Serbia (two sites), and Turkey.

### Study design

Volunteer interdisciplinary teams underwent 2–3 simulated scenarios of medical emergencies prior to clinical implementation of the CERTAIN platform and prior to completing the CERTAIN-ELITE training. They were subsequently given access to online training tools with remote coaching and support [9]. After the 2–4-week training sessions of online tutorials and remote coaching, subjects underwent an additional 2–3 simulated scenarios. Pre- and post-CERTAIN training scenarios were recorded by the training team via video conference software (Google Hangout and Zoom) and uploaded to a secure YouTube Channel.

The pre- and post-CERTAIN training simulation were evaluated by reviewers at a remote site. The first pre- and post-CERTAIN training simulations were excluded as an ‘adjustment’ period. The remaining pre- and post-CERTAIN training simulation recordings were evaluated by two independent, medically trained reviewers utilizing the TEAM assessment tool [11]. The TEAM assessment tool has been validated in the intensive care unit as a method to assess team dynamics [11]. The pool of reviewers (five medically trained reviewers) were introduced to the TEAM assessment tool and given an overview of the rating system. Then reviewers watched three simulations and independently assessed utilizing the TEAM assessment tool. Reviewers were de-briefed and consensus grading was reached. For simulation grading, reviewers were then chosen at random from the pool of reviewers. The reviewers were blinded to the status of the training team’s completion of CERTAIN modules. Any discrepancies of greater than 1 point between the two reviewers on any assessment question, excluding the global team rating, were sent to a third reviewer from the original pool. The score that was deemed discordant by the third reviewer was eliminated and replaced with the score of the third reviewer. The global team rating score given by the initial two reviewers was maintained for all simulations.

### Data analysis

Pre- and post-CERTAIN training TEAM scores were averaged for each individual assessment and compared using Wilcoxon Matched-Pairs Signed-Ranks Test (*p*-values ≤0.05 were considered statistically significant). Changes in scores were calculated as absolute values and a percent of the pre-training scores with negative scores representing a decrease in average score. Statistical analysis was performed on SPSS software (IBM Corp. Released 2010. IBM SPSS Statistics for Windows, Version 19.0. Armonk, NY: IBM Corp).

## Results

Thirty-four videos were reviewed (17 pre-CERTAIN training and 17 post-CERTAIN training). Fifteen (44.1%) simulations required a third reviewer secondary to discrepancies of greater than 1 point on any of the 11 team assessment questions. Twelve out of 15 reviews differed on 1 out of 11 questions. The remaining 3 reviews differed on 2 or more questions.

For each of the 11 TEAM assessment areas, a trend toward improvement following CERTAIN training was noted. No individual assessment had universal improvement (supplemental table, Table S1). Average absolute improvement for each of the 11 TEAM assessments ranged from 0.1 to 0.7. ‘Team composure and control’ had the least absolute score improvement (2.5–2.6, *p* = 0.89). The greatest average improvement was in the ‘team’s ability to complete tasks in a timely manner’ and in the ‘team leader’s communication to the team’. Six out of nine teams demonstrated an improvement in these areas with an average improvement from 2.1 to 2.7 (28.5%, *p* = 0.05) and 2.3 to 3.0 (30.4%, *p* = 0.03), respectively. ‘Team task prioritization’ and ‘Team reassessment of the clinical situation’ also demonstrated statistically significant improvement (*p* = 0.05 and *p* = 0.02, respectively). Average global team assessment improved from 5.8 to 6.9 (*p* = 0.04) following CERTAIN remote training.

Six teams demonstrated an overall improvement in global team assessment scores following CERTAIN training (Table 1). These teams demonstrated an improvement of 0.4–4.3 points in their global assessment scores with five teams improving their scores by more than 10%. Of the remaining three teams, one team maintained the same global team assessment score (Turkey), and the other two teams decreased their global team assessment score by 6% or less (Ireland and Serbia 2).

**Table 1.** Average global assessment score for each team pre and post CERTAIN training. The change in score is expressed as a percent of the pre-training score. The global assessment is determined a 1–10 Likert scale with 10 representing aspirational team performance.

Team	Pre/post CERTAIN training	Global assessment score average	Percent change in global assessment score
Bosnia	Pre	5.3	7.0
	Post	5.7	
Brazil	Pre	4.8	36.0
	Post	7.5	
India	Pre	5.0	20.6
	Post	6.3	
Ireland	Pre	8.5	−2.4
	Post	8.3	
Mexico	Pre	7.0	10.3
	Post	7.8	
Saudi	Pre	4.0	51.8
Arabia	Post	8.3	
Serbia	Pre	5.5	19.1
	Post	6.8	
Serbia 2	Pre	5.3	−6.0
	Post	5.0	
Turkey	Pre	6.5	0
	Post	6.5	

## Discussion

Most teams demonstrated an overall improvement following remote simulation (six out of nine teams), and all teams demonstrated improvement in at least one area. The greatest average area of improvement was in the ‘team’s ability to complete tasks in a timely manner’ and in the ‘team leader’s communication to the team’. The team’s ability to complete tasks in a timely manner likely increased because of the availability of a structured tool (CERTAIN-ELITE) that prompted the team as to the next step in goal-directed resuscitation. This likely impacted the communication amongst team members, which is reflected in the improved overall performance in these two areas. The teams who did not demonstrate overall improvement were Ireland, Serbia 2, and Turkey. Ireland had the highest pre-CERTAIN training performance, and the lack of improvement following training may be a reflection of their already high performance leaving less room for improvement. Serbia 2 and Turkey appeared to have difficulty with team communication. Since all simulations were performed in English, we feel their lack of improvement may have been secondary language barriers amongst team members and reviewer difficulty in interpreting team member communications. Moreover, team leaders rotated for all simulations, which may have had a negative impact on their performance (i.e., if a ‘weak’ team member assumed the team leader role for the post-CERTAIN-ELITE simulations). On the same front, scores may be falsely elevated for teams if a ‘strong’ team member assumed the team leader role for the post-CERTAIN-ELITE simulations.

Mannequin based and standardized patient simulation improved clinical skills and performance [2,5]. Prior studies have evaluated the feasibility of virtual simulation and the effects of simulation on teamwork [2,3], and these studies have found a positive effect on team dynamics via these simulation methods, similar to our study. To our knowledge, no studies have examined the feasibility of long-distance, remote simulation to improve teamwork.

Low-cost simulation and utilization of tools already available to trainees and teams were strengths of the study. Limitations of the study included language/cultural barriers and video quality. All teams completed the training modules in English. This may have affected the teams’ overall communication and performance scores. Ireland was the only team with English as a first language, and this may have influenced their high-performance scores. Moreover, while the TEAM assessment tool has been validated in the intensive care unit, it has not been directly validated as a tool to measure team dynamics in remote simulation. The teams evaluated during the simulation settings consisted of a variety of practitioners at different levels of training; however, the demographic information including age and sex is not known. The participants’ age and prior experience with technology may have positively or negatively impacted the results.

In conclusion, the assessment of team dynamics using remote simulation appears to be feasible and results in improved team performance. To further evaluate these findings, larger studies with more simulation groups are needed with the addition of team self-assessments.
