# Supplementary material for: Evaluation of TEAM dynamics before and after remote simulation training utilizing CERTAIN platform
Source: Med Educ Online. 2018 Jun 18;23(1):1485431. doi: 10.1080/10872981.2018.1485431 (PMC6008595; doi:10.1080/10872981.2018.1485431)
Supplement: Supplemental Material [file ZMEO_A_1485431_SM7071.docx]

| Location: | Pre or Post Simula-tion | Team Leader commun-ication | Team Leader Pers-pective | Team commun-ication | Team task comple-tion | Team compos-ure | Team morale | Team adap-ted | Team reass-ess-ment | Team anticipa-tion | Task prior-itization | Followed standards | Team global rating |
| --- | --- | --- | --- | --- | --- | --- | --- | --- | --- | --- | --- | --- | --- |
| Bosnia | Pre | 2.0 | 2.0 | 2.0 | 1.8 | 2.8 | 2.8 | 2.0 | 1.8 | 2.0 | 2.0 | 1.8 | 5.3 |
|  | Post | 2.5 | 1.8 | 1.8 | 2.8 | 1.8 | 1.8 | 2.0 | 2.3 | 2.0 | 2.0 | 2.8 | 5.8 |
| Brazil | Pre | 1.5 | 2.5 | 1.5 | 1.5 | 2.5 | 2.3 | 2.5 | 2.3 | 1.8 | 2.0 | 2.0 | 4.8 |
|  | Post | 3.0 | 3.3 | 3.5 | 2.5 | 2.0 | 3.3 | 1.8 | 2.5 | 2.3 | 2.3 | 2.8 | 7.5 |
| India | Pre | 2.3 | 1.3 | 1.5 | 1.5 | 1.8 | 2.0 | 1.0 | 1.0 | 1.5 | 1.5 | 1.8 | 5.0 |
|  | Post | 2.8 | 2.3 | 2.3 | 1.5 | 2.8 | 3.3 | 1.8 | 2.8 | 2.8 | 2.3 | 2.3 | 6.3 |
| Ireland | Pre | 3.3 | 3.3 | 3.3 | 3.5 | 3.0 | 3.0 | 3.0 | 3.0 | 3.3 | 3.3 | 2.5 | 8.5 |
|  | Post | 3.3 | 3.0 | 3.3 | 3.0 | 3.3 | 3.3 | 3.3 | 3.3 | 3.3 | 3.0 | 3.0 | 8.3 |
| Mexico | Pre | 3.3 | 3.3 | 3.3 | 3.0 | 3.3 | 4.0 | 3.0 | 3.0 | 3.0 | 2.8 | 2.8 | 7.0 |
|  | Post | 3.3 | 3.3 | 3.3 | 3.3 | 3.5 | 3.5 | 2.8 | 3.8 | 3.0 | 3.3 | 2.5 | 7.8 |
| Saudi Arabia | Pre | 1.5 | 2.0 | 1.5 | 1.0 | 2.0 | 1.8 | 1.8 | 2.3 | 1.8 | 2.0 | 2.0 | 4.0 |
|  | Post | 3.3 | 3.0 | 3.3 | 3.0 | 3.0 | 3.5 | 3.3 | 3.3 | 3.0 | 3.3 | 3.0 | 8.3 |
| Serbia | Pre | 2.3 | 2.0 | 1.5 | 1.8 | 2.5 | 2.3 | 2.3 | 2.8 | 2.0 | 2.0 | 2.3 | 5.5 |
|  | Post | 3.0 | 3.0 | 3.0 | 3.0 | 2.8 | 3.0 | 2.3 | 2.8 | 3.3 | 3.0 | 2.8 | 6.8 |
| Serbia 2 | Pre | 1.8 | 1.5 | 2.0 | 2.3 | 2.5 | 2.0 | 2.0 | 2.3 | 2.0 | 1.8 | 2.5 | 5.3 |
|  | Post | 3.0 | 2.8 | 2.3 | 2.8 | 2.0 | 2.5 | 2.0 | 2.5 | 1.8 | 2.0 | 2.3 | 5.0 |
| Turkey | Pre | 2.5 | 2.5 | 3.0 | 3.0 | 2.5 | 3.0 | 2.5 | 3.0 | 2.5 | 2.5 | 3.0 | 6.5 |
|  | Post | 2.5 | 2.5 | 2.5 | 3.0 | 2.5 | 2.5 | 3.0 | 3.0 | 2.0 | 2.5 | 2.5 | 6.5 |
| **Avg** | **Pre** | **2.3** | **2.3** | **2.2** | **2.1** | **2.5** | **2.6** | **2.2** | **2.4** | **2.2** | **2.2** | **2.3** | **5.8** |
|  | **Post** | **3.0** | **2.8** | **2.8** | **2.8** | **2.6** | **2.9** | **2.6** | **2.9** | **2.6** | **2.6** | **2.6** | **6.9** |
| **P value** |  | **0.03** | **0.06** | **0.09** | **0.05** | **0.78** | **0.23** | **0.25** | **0.02** | **0.14** | **0.05** | **0.07** | **0.04** |
